# “Breaking news” from spermatids

**DOI:** 10.1186/2051-4190-23-11

**Published:** 2013-11-07

**Authors:** Anne Gouraud, Marc-André Brazeau, Marie-Chantal Grégoire, Olivier Simard, Julien Massonneau, Mélina Arguin, Guylain Boissonneault

**Affiliations:** Dept of Biochemistry, Faculty of Medicine and Health Sciences, Université de Sherbrooke, Pavillon Z8, 3201 Jean-Mignault St, Sherbrooke, Quebec J1E 4K8 Canada

**Keywords:** Spermiogenesis, Chromatin remodeling, DNA break, Torsional stress, Topoisomerase, Apoptosis, Oxidative stress, Spermiogenèse, Remodelage de la chromatine, Cassures de l’ADN, Stress torsionnel, Topoisomérase, Apoptose, Stress oxidatif

## Abstract

During the haploid phase of spermatogenesis, spermatids undergo a complex remodeling of the paternal genome involving the finely orchestrated replacement of histones by the highly-basic protamines. The associated striking change in DNA topology is characterized by a transient surge of both single- and double-stranded DNA breaks in the whole population of spermatids which are repaired before spermiation. These transient DNA breaks are now considered part of the normal differentiation program of these cells. Despite an increasing interest in the study of spermiogenesis in the last decade and the potential threat to the haploid genome, the origin of these DNA breaks still remains elusive. This review briefly outlines the current hypotheses regarding possible mechanisms that may lead to such transient DNA fragmentation including torsional stress, enzyme-induced breaks, apoptosis-like processes or oxidative stress. A better understanding of the origin of these DNA breaks will lead to further investigations on the genetic instability and mutagenic potential induced by the chromatin remodeling.

## Résumé

Lors de la phase haploïde de la spermatogenèse, les spermatides subissent un remodelage complexe du génome paternel impliquant un remplacement finement orchestré des histones par des protamines hautement basiques. Le changement topologique de l’ADN associé est caractérisé par une augmentation transitoire de cassures simple et double brins de l’ADN dans l’entière population des spermatides qui sont réparées avant la spermiation. Ces cassures transitoires de l’ADN sont maintenant considérées comme faisant partie du processus normal de différenciation de ces cellules. Malgré un intérêt croissant dans l’étude de la spermiogenèse ces 10 dernières années et la menace potentielle pour le génome haploïde, l’origine de ces cassures d’ADN reste encore incertaine. Cette revue décrit brièvement les hypothèses actuelles concernant les mécanismes possibles qui pourraient mener à cette fragmentation transitoire de l’ADN incluant le stress torsionnel, les cassures enzymatiques, des processus semblables à l’apoptose et le stress oxidatif. Une meilleure compréhension de l’origine de ces cassures d’ADN mènerait à des études approfondies concernant l’instabilité génétique et le potentiel mutagène induit par le remodelage de la chromatine.

## Introduction

In addition to the reduction of their chromosome number, post meiotic haploid cells (spermatids) undergo one of the most drastic changes in nuclear organization known to the eukaryotic domain. The male gamete’s genome is compacted six fold into the head of the spermatozoon within a nucleus about 40 times smaller than a somatic cell [1]. Proper compaction is essential for fertilization and embryogenesis [2] and alteration in the sperm chromatin state is linked to impaired spermatogenesis and infertility [3, 4]. The sperm chromatin state is mainly determined during spermatids’ differentiation (spermiogenesis) resulting from the sequential replacement of the majority of histones by transition proteins and protamines, which are highly basic arginine-rich proteins. Removal of histones is likely facilitated by hyperacetylation [5–9], leading to a more open chromatin state [10]. From studies using histone deacetylases inhibitors, induced hyperacetylation of histones in cultured cells leads to a greater vulnerability to DNA damage and DNA double-strand breaks (DSBs) are observed [11, 12]. Following histone withdrawal, the protaminated DNA adopts a completely different topology as the somatic-like DNA supercoiling is lost and planar toroids are formed [1]. As shown by TUNEL assay performed in both human and mouse, the whole population of spermatids undergoes transient DNA breaks. In this case, labeling by the terminal transferase indicates that 3’OH DNA ends are generated [13]. One hypothesis is that the formation of DNA breaks would be necessary to relieve the torsional stress induced by the accumulation of free supercoils. Most interestingly, recent evidence from our group and others indicate that a significant proportion of these breaks are double-stranded [14, 15]. DSBs represent a much greater genetic threat for the cell [16, 17], especially in a haploid context such as in spermatids. Due to the lack of sister chromatid, templated homologous recombination cannot proceed and only error-prone end-joining mechanisms may operate. For this reason, the origin, localization, as well as the pathway involved in the repair process represent a growing interest in the field of reproductive genetics as this may represent a major component of the male mutation bias that have now been confirmed in recent studies [18–20]. Of interest is the propensity for DNA end-joining mechanism in haploid context to generate insertion/deletions that have been reported to represent the most severely male-biased type of male mutations [21, 22].

The mechanism involved in the transient formation of DNA strand breaks has yet to be identified. This brief review focuses on three potential mechanisms likely to induce DNA breaks during the chromatin remodeling process. It also cannot be excluded that a combination of such mechanisms may be involved.

### Torsional stress

One hypothesis for the formation of DNA breaks during spermiogenesis is the simple increase in torsional stress resulting from the accumulation of free supercoils. Before chromatin remodeling, most of the DNA is wound around histone octamers in almost two superhelical turns as in somatic cells [23]. In doing so, nucleosomes constrain the potential energy of supercoiling. However, when histones are withdrawn from spermatid’s chromatin, the density of free supercoils (torsional stress) must increase rapidly. In fact, torsional stress is strong enough to alter proteins-DNA interaction and regulate transcription [24, 25]. For example, in absence of DNA relaxing proteins such as topoisomerase I, the DNA polymerase barely progresses more than 300 pb due to the accumulation of torsional stress [26]. An increase in torsional stress may induce local denaturation of DNA especially at AT-rich regions [25]. Moreover, the accumulation of free negative supercoils during chromatin remodeling could also change the conformation of the DNA, from a canonical B-DNA helix to non-B DNA structures, such as cruciform, hairpin or left-handed Z-DNA [27, 28]. These non-canonical DNA conformations are more likely to produce DNA breakage leading to genetic instability often associated with translocations [29, 30]. It is however unlikely that this process alone is responsible for the majority of the DNA breaks that are observed as these “mechanical” breaks are unlikely to produce the free 3’OH DNA ends, as revealed by the TUNEL assay.

### Enzymatic pathway

#### Topological enzymes

As stated above, detection of free 3’OH by the *in situ* TUNEL assay strongly suggests that DNA strand breaks in spermatids are likely to result from an enzymatic process. As a major change in topology is observed during spermiogenesis, enzymes specialized in changing the topology of DNA become good candidates. These enzymes are termed topoisomerases and are grouped in two different classes namely type I and II, depending on whether they change the linking number in steps of one or two respectively [31]. Although both topoisomerases IIA (TOP2A) and IIB (TOP2B) are known to be expressed in testis, TOP2A is not detected in elongating spermatids [32], whereas TOP2B is present during chromatin remodeling steps [33]. However, if topoisomerases were indeed generating the observed DNA strand breaks, the terminal deoxynucleotidyl transferase (TdT) would not be able to gain access to any topoisomerase-induced DNA breaks due to the double covalent binding of TOP2B on both 5'-phosphate DNA ends [34] that is expected to create steric hindrance at the 3’OH therefore preventing the TUNEL labeling. Interestingly, results from our group indicate that expression of TDP1, a phosphodiesterase responsible for the removal of both TOP1-DNA and TOP2-DNA adducts [35], is coincident with TOP2B expression during the spermatid's elongation process [33]. Although TDP2 is known to preferentially interact with TOP2B, its expression has not yet been investigated during spermiogenesis. In addition, a recent study indicated that TOP2B activity could be inhibited by Poly(ADP-Ribose) Polymerases 1 and 2 (PARP1/2), which are themselves activated following TOP2B-induced DSB. Increase of poly(ADP-ribosyl)ation at break sites might cause early release of TOP2B, since it has lost its ability to bind DNA due to its interaction with the highly negatively charged polymer [36]. According to this observation, one may surmise that DSBs could be a direct result of an abortive TOP2B catalytic cycle during chromatin remodeling in elongating spermatids.

During meiosis, the SPO11 enzyme catalyzes the formation of DSBs required for homologous recombination [37]. SPO11 forms a dimer and is related to an archeal topoisomerase VI. SPO11 interacts with DNA in a similar manner to TOP2B, covalently binding the 5'-phosphate DNA ends but leaving a free 3'OH nucleotide because of an upstream endonuclease activity [38]. The free 3’OH then becomes available for TdT labeling [37]. Hence the possibility exists that this meiotic topoisomerase-like enzyme could be involved in the formation of transient DSBs observed in spermatids. Interestingly, round spermatid expression data indicate that SPO11 transcripts are present to a much higher level than those of TOP2B during early spermiogenesis [39]. This therefore warrants further investigation regarding the expression and the functional role of the SPO11 proteins in the formation of chromatin remodeling DSBs.

#### Apoptosis-like pathway

Terminal differentiation of vertebrate cells, including lens fiber cells and erythrocytes appears molecularly and biochemically related to apoptosis [40]. Interestingly, spermatids’ differentiation also shares similarities with apoptosis being characterized by cytoplasmic extrusion [41], chromatin condensation [42] and phosphorylation of the histone variant H2AX [43]. Preliminary mass spectrometry data on spermatidal nuclear proteins generated by our group (Leroux *et al*., unpublished) have shown expression of some apoptotic proteins during the chromatin remodeling in spermatids. During apoptosis, the initial DNA fragmentation is induced by a caspase-activated endonuclease known as the DNA fragmentation factor subunit β (DFFB) which produces blunt-ended 3'OH termini [44]. Interestingly, this nuclease is expressed during spermatogenesis [45] and is known to be stimulated by TOP2B, HMGB1/2 and histone H1 when added to nucleosome-free DNA [46]. Such a transient state of “open” chromatin is indeed found during spermiogenesis. DFFB deserves further investigation as a candidate endonuclease that may be responsible for the observed DNA fragmentation pattern in elongating and condensing spermatids. It is likely however that such DNA cleavage activity is a finely regulated process since most spermatids seemingly survive this genetic insult and most DNA strand breaks become repaired at later steps. Finally, new apoptosis-like pathway may be used in the testis as both RNA splicing and proteolysis are especially active in this tissue. For instance, the RNAse DICER is essential for normal chromatin organization and nuclear shaping of spermatids [47]. In *C. elegans*, DICER could be cleaved by the action of CED-3 caspases, resulting in a truncated C-terminal fragment (tDCR-1) with a new DNase activity instead of its normal RNase activity [48]. Investigations regarding the involvement of a potential truncated variant of DICER in the generation of the transient surge of DNA breaks in spermatids are underway. Other evidence of apoptosis-like process were also shown during spermatid individualization in *D. melanogaster*[49]. In addition, interleukin-1β-Converting Enzyme (ICE), a mammalian ortholog for CED-3 caspase, was also found in residual bodies in rat testis [50, 51]. Taken together, these evidence are pointing to the possibility of a controlled apoptosis-like mechanism able to generate reversible DNA fragmentation in spermatids. The purpose of this remains unclear but one hypothesis is that the non-templated repair process that is required to reverse fragmentation would be a newly identified mechanism for the generation of transmittable *de novo* genetic polymorphism since end-joining repair processes are error-prone [52].

#### Oxidative stress

Several studies have highlighted the role of oxidative stress in sperm DNA damage and male infertility [53, 54]. Indeed, reactive oxygen species (ROS), which are mostly byproducts of oxygen metabolism occurring in mitochondria, are associated with the formation of apurinic/apyrimidique (AP) DNA sites, oxidized purines or pyrimidines and DNA breaks in the mature spermatozoa. AP sites and oxidized bases are principally related to mutagenesis, but the direct interaction of hydroxyl radicals with DNA can induce single-strand DNA breaks (SSBs). More precisely, a DNA break can be created by an OH-mediated hydrogen abstraction at C3’, C4’ and C5’ of the 2-deoxyribose [55, 56]. The attack of DNA by several hydroxyl radicals could generate adjacent SSBs and possibly DSBs. Nevertheless, little is known about the implication of ROS during spermiogenesis. As the chromatin is in a more open configuration during the remodeling occurring in spermatids, one can hypothesize that the DNA become transiently vulnerable to DNA-damaging agent such as ROS. Not surprisingly, spermatozoa with an incomplete protamination, resulting in a less condensed DNA, have been shown to be more sensitive to oxidative stress [57].

The primary known source of ROS is the electron transport chain in mitochondria. Although enzymatic production of ROS by a NADPH oxidase (NOX) needs further investigations, it is tempting to speculate that the recently found association of NOX5 mRNAs with the plasma membrane of round spermatids in human and equine testis [58, 59] shows the potential for elongating and condensing spermatids to produce ROS, since a large fraction of mRNAs are translationally repressed in round spermatids and translated at later steps [60]. It is also noteworthy that ROS are indispensable for mechanisms such as capacitation, acrosome induction and fertilizing ability of spermatozoa [61]. ROS activity may therefore extend to the induction of breaks in the DNA of spermatids. The quantification of 8-oxo guanine, used as a reliable oxidative stress marker [62], during the spermatid’s chromatin remodeling steps could be an interesting way to investigate the implication of ROS at these stages.

## Conclusion

Transient DNA breaks are normal and probably a necessary mechanism for the differentiation of spermatids as they are likely to facilitate the nucleosome-to-protamine transition by elimination of the free DNA supercoils. The transient increase in DNA strand breaks may result from one or a combination of the mechanisms described above. This process must also be finely regulated since an excess of DNA fragmentation may lead to true apoptosis or the persistence of DNA breaks in the mature sperm. Such persistence of DNA strand breaks resulting from alterations in chromatin remodeling may be one of the leading cause of increased sperm DNA fragmentation associated with subfertility [54, 63].

## Abbreviations

DSB: Double-strand break
TOP2A: Topoisomerase IIA
TOP2B: Topoisomerase IIB
TdT: Terminal deoxynucleotidyl transferase
DFFB: DNA fragmentation factor subunit β
ROS: Reactive oxygen species
SSB: Single-strand break
NOX: NADPH oxydase.
